# Effects of short-term feeding with high fiber diets on growth, utilization of dietary fiber, and microbiota in pigs

**DOI:** 10.3389/fmicb.2022.963917

**Published:** 2022-07-27

**Authors:** Guang Pu, Liming Hou, Taoran Du, Binbin Wang, Hang Liu, Kaijun Li, Peipei Niu, Wuduo Zhou, Ruihua Huang, Pinghua Li

**Affiliations:** ^1^Key Laboratory of Pig Genetic Resources Evaluation and Utilization, Ministry of Agriculture and Rural Affairs, Institute of Swine Science, College of Animal Science and Technology, Nanjing Agricultural University, Nanjing, China; ^2^Laboratory of Intestinal Microbiology, Huaian Academy, Nanjing Agricultural University, Nanjing, China; ^3^Industrial Technology System Integration Innovation Center of Jiangsu Modern Agriculture (PIG), Nanjing, China

**Keywords:** pigs, short-term feeding, fiber intake, growth performance, fiber-degrading microbiota

## Abstract

Finishing pigs can adapt to high-fiber diet smoothly according to the production performance and their intestinal microbiota through a 28-day trial or longer. However, it is unclear, at which stage during the experimental period, the adaptation occurred. Here we studied the dosage effects of dietary fiber (Total dietary fiber (TDF) from 16.70 to 24.11%) on growth performance, fiber digestibility, fecal microbiota, and microbial fermentation of finishing pigs during a 14-day feeding period. The results showed that the average daily feed intake (ADFI) and feed/gain (F/G) of pigs were not affected as the dietary fiber increased. Apparent total tract digestibility (ATTD) of cellulose, hemicellulose, insoluble dietary fiber (IDF), soluble dietary fiber (SDF), and TDF of pigs remained unchanged when TDF was between 16.70 and 17.75%, while strikingly decreased when TDF increased from 17.75 to 24.11%. It is worth noting that increasing fiber intake seemed to favor hemicellulose digestion. In addition, the increase in fiber intake increased fecal microbial diversity, especially improved the proportion of the members of the family Prevotellaceae, Ruminococcaceae, and Lachnospiraceae, and decreased the abundance of the genus *Streptococcus*. Moreover, the increase in fiber intake promoted the digestion of fiber, production of short chain fatty acids (SCFAs), and enhanced microbial pyruvate metabolism and butanoate metabolism. In conclusion, short-term high fiber feeding has no adverse effects on the growth performance of finishing pigs. ATTD of dietary fiber of finishing pigs was maintained when TDF was at 17.75%. And short-term high fiber feeding improved microbial diversity and fiber degradation functions of finishing pigs.

## Introduction

Currently, a lot of fiber-rich ingredients were widely added to the diets of livestock, which also included pigs. However, various studies found that diets with a high fiber content could restrain the nutrient digestibility and feed intake in growing pigs (Kyriazakis and Emmans, 1995; Wilfart et al., 2007a; Zhang et al., 2013). The relative advantages and disadvantages of adding fiber to pig diets depend on the stage of pig production. Stewart et al. (2013) found that the impact of the inclusion of dietary fiber depends on the maturity of the animals. Dietary fiber may have little impact on energy retention in finishing pigs, while it could compromise the ability to grow pigs to obtain adequate energy. Thus, it is necessary to study the suitable dosage of dietary fiber without damaging the growth and nutrient digestibility of pigs at specific growth stages.

It is well known that dietary fiber plays a positive role in maintaining the diversity of gut microbiota and intestinal development in both humans and pigs (Knudsen, 2001; Jha et al., 2010). A high-fiber diet was also related to the increased amount of fiber-degrading-related microbial taxa in the swine large intestine (Varel, 1987; Metzler and Mosenthin, 2008). This increased amount of fiber-degrading-related microbial taxa favors the colonization and growth of beneficial bacteria, meanwhile, which is beneficial for the overall host intestinal health (Williams et al., 2002), whereas suppresses the harmful ones. Several bacterial taxa such as *Oscillibacter*, Christensenellaceae, *incertae sedis*, Defluviitaleaceae *Corynebacterium*, and *Cellulosilyticum* were reported to be positively associated with feed efficiency (McCormack et al., 2017) and critical for the swine industry. Recent studies have also filled some knowledge gaps of the swine gut microbiome, with respect to the biogeography of the gastrointestinal tract (Van Hees et al., 2019), digestibility (He et al., 2018), and growth performance (Tsai et al., 2018). However, it is still not clear how gut microbiota correlates with the growth and fiber utilization of pigs.

It takes time for pigs to adapt to the new fermentation substrate and fiber, and for their microbiota to reach a relatively stable Stage. Martinez-Puig et al. (2003) estimated that the gastrointestinal tract of growing pigs took 5 weeks to adapt to a raw potato starch diet, as assessed by fecal excretion and whole tract digestibility. However, others found that pigs can produce some positive responses to short-term high fiber feeding, including the maintenance of pig growth performance and the optimization of intestinal microbial composition. For instance, during a 21-day animal trial, Zhang et al. (2019) added 7.14% oat β-glucan and 5.05% microcrystalline cellulose in the basal diet separately for growing Duroc × Landrace × Yorkshire barrows and found that these fiber additions had no effects on growth performance of pigs, while significantly changed the microbial composition of the hindgut. Haenen et al. (2013) found diet containing 34% resistant starch induced the stimulation of the butyrate-producing *Fecalibacterium prausnitzii*, meanwhile, reducing the proportion of potentially pathogenic members of the *Gammaproteobacteria*, including *Pseudomonas spp*. and *Escherichia coli* after 2 weeks. Similarly, a 2-week feeding study revealed that pigs fed the freeze-dried kiwifruit or kiwifruit fiber diets had a lower number of *E. coli* and *Enterobacteria* and a significantly higher number of total bacteria and *Bacteroides* group, as well as a higher ratio of *Lactobacillus* to *Enterobacteria* when compared to pigs fed the basal diet (Han et al., 2011). These studies suggested that high fiber diet could modulate the intestinal microbiota of pigs even during a relative short-term feeding.

Chinese Suhuai pig is a new lean-type pig breed, inheriting 25% Chinese indigenous Huai pig ancestry and 75% Large White ancestry, and possesses excellent tolerance to high fibrous feedstuffs (Zhang Y. Q. et al., 2016; Wang B. et al., 2019). Defatted rice bran (DFRB) is the by-product of the defatting treatment of whole fat rice bran. It has high dietary fiber content (Supplementary Table S1) and is a good fiber raw material (Wan et al., 2012). In addition, the yield of DFRB in China is tremendous, and DFRB has been widely used in the feeding of Chinese native pigs. Now in China, in order to reduce feed costs, DFRB will also be used in lean pig breeds. But the high fiber content of DFRB will lead to its low utilization rate. It is particularly important to study the appropriate proportion of DFRB in feed. Therefore, DFRB was used as a main fibrous source to study the response of Chinese Suhuai pigs to a high fiber diet in the current study. A previous study found that the Suhuai pig could adapt to the diet containing 19.15% TDF after 28 days of feeding, which is mainly reflected in the maintenance of growth performance, apparent total tract digestibility (ATTD) of fiber, and the increase of abundance of caecal fiber-degrading bacteria (Pu et al., 2020). Congeneric studies have shown that pigs show adaptation to a high fiber diet in a 14-day high fiber feeding (Han et al., 2011). However, there was limited information about the effect of short-term high fiber feeding on fiber digestibility and intestinal microbial composition of Suhuai pigs.

Consequently, we hypothesized that in the early stage of the feeding experiment, Suhuai pigs had an adaptation to a high fiber diet, and this adaptation might be accompanied by changes in the intestinal microbial structure. Therefore, based on the previous experimental design and animals, growth performance and ATTD of fiber on day 14 of the experimental period were measured to evaluate the responses of Suhuai finishing pigs to a high fiber diet. In addition, the fecal microbial community and functions of Suhuai finishing pigs on day 14 of the experimental period were analyzed to explore the adaptability of intestinal microbiota to a high fiber diet in a short-term feeding period.

## Materials and methods

### Ethics statement

All animal trials were performed following Guidelines for the Care and Use of Laboratory Animals prepared by the Institutional Animal Welfare and Ethics Committee of Nanjing Agricultural University, Nanjing, China [Certification No: SYXK (Su) 2017-0007].

### Experimental design and sample collection

The details of experimental design, selection of experimental animals, animal feeding, and management have been presented in our previous study (Pu et al., 2020). Briefly, a total of 35 Suhuai barrows with a body weight of 62.90 ± 0.78 kg were selected and allotted into five groups: the control group, and treatment I-IV using a completely randomized design. All pigs were fed by the Osborne Testing Stations System (OTSS, provided by OSB Livestock Technology Co., Ltd. Shanghai, China), which can accurately record daily intake, and body weight individually. So, each pig is identified as a replicate, and hence seven replicates in each group. During the pre-feeding period of 10 d, all pigs were fed with the basal diet. During the 14 days of the trial period, the pigs in the control group and treatment I–IV were provided with diets: the basal diet (the same basal diet used in the pre-feeding period), 7, 14, 21, and 28% of DFRB (as feed basis) substituted equivalent corn respectively. The composition and nutritional level of five experimental diets were shown in Supplementary Table S1, and the nutritional level of DFRB and corn was shown in (Supplementary Table S2). On day 14 of the trial, the fecal sample of each pig was collected. At least 200 g of feces per pig were collected. The collection method was described as follows.

When the pig was defecating or was about to defecate, one person quickly caught the feces with a self-zip plastic bag so that the feces do not touch the ground. Meanwhile, another person labeled the pig with spray lacquer. Through the above fecal collection methods, we can trace the fecal samples and the pig's ID clearly, and ensure that the fecal sample of each pig was in order. Before sampling, tweezers, 2 ml cryopreservation tubes, and gloves used to collect microbial samples were autoclaved and dried to ensure that all tools were sterile. Fecal uncontaminated samples at the fecal center location were collected into sterile tubes and stored at −80°C for 16S rRNA sequencing, and determined the activity of fiber degrading enzyme and short chain fatty acids (SCFAs), then the rest of the feces were collected and stored at −20°C for chemical analysis.

### Growth performance analysis

Based on the data of daily intake and body weight from day 1 to day 14. Then the results of average daily feed intake (ADFI), average daily gain (ADG), and feed gain ratio (F/G) were calculated.

### ATTD of dietary components analysis

Naturally occurring acid-insoluble ash (AIA) of diets and feces was used as an endogenous marker for calculating ATTD of dietary components in this study (McCarthy et al., 1974). About 200 g of feces or diets per sample were fully dried (100°C for 4 h). About 2 g (2.0000–2.0050 g) diets and fecal samples were accurately weighed for AIA analysis. Three technical replicates per sample. Diets and fecal samples were analyzed for DM [Method 930.15; AOAC (2007)], diets and ingredients were also analyzed for AIA [Method 942.05; AOAC (2007)], and IDF and SDF in diets and ingredients were determined using the Ankom TDF Dietary Fiber Analyzer [AOAC 991.43, AOAC (2007); Ankom Technology, Macedon, NY].

### DNA extraction, PCR amplification, and illumina MiSeq sequencing

Based on the difference in ATTD of fiber among five groups, the samples from the control group, treatment I, treatment II, and treatment IV were selected for 16S gene sequencing to explore their differences in fecal microbial composition. Total microbial genomic DNA was extracted from fecal samples using the E.Z.N.A.^®^ soil DNA Kit (Omega Bio-tek, Norcross, GA, U.S.) according to the manufacturer's protocols. The V3-V4 region of the bacterial 16S rRNA gene was amplified by PCR using bacterial universal primers (341F 5′-AGAGTTTGATCCTGG CTC AG-3′ and 806R 5′-TTACCGCGGCTGCTGGCAC-3′) (Claesson et al., 2010). Purified amplicons were pooled in equimolar and 2 ×300 paired-end libraries were sequenced on an Illumina MiSeq platform according to the standard protocols at the Majorbio Bio-Pharm Technology Co., Ltd. (Shanghai, China).

The sequencing raw data were quality-filtered by Trimmomatic and merged by FLASH with the following criteria: (i) The reads were truncated at any site receiving an average quality score <20 over a 50 bp sliding window. (ii) Sequences whose overlap was longer than 10 bp were merged according to their overlap with a mismatch of no more than 2 bp. (iii) Sequences of each sample were separated according to barcodes (exactly matching) and Primers (allowing 2 nucleotide mismatching), and reads containing ambiguous bases were removed. Operational taxonomic units (OTUs) were clustered with a 97% similarity cutoff using UPARSE (version 7.1 http://drive5.com/uparse/) with a novel “greedy” algorithm that performed chimera filtering and OTU clustering simultaneously. The taxonomy of each 16S rRNA gene sequence was analyzed by the RDP Classifier algorithm (http://rdp.cme.msu.edu/) against the Silva (SSU123) 16S rRNA database using a confidence threshold of 70% (Wang et al., 2019a).

### Activity of fiber degrading enzyme analysis

About 150 ml of 1% (w/v) solution of D-Salicin, microcrystalline cellulose, carboxymethyl cellulose, and filter papers were used as the reaction substrate. Cellulase Kit (Nanjing Jiancheng Bioengineering Institute of China, Nanjing, Jiangsu, China) was used to extract crude enzymes. Briefly, weighed about 0.1 g frozen feces, added 1 ml buffer, after homogenized feces, centrifuged feces (8,000 × g for 10 min at 4°C. Then the crude enzyme (supernatant) was acquired. A 50 μl crude enzyme solution extracted from each fecal sample was added to determine the activity of microcrystalline cellulase, salicinase, carboxymethyl cellulase, and filter paper enzyme using Microplate Reader at 600 nm (OD_600_), respectively.

### Determination of fecal SCFAs

The SCFAs concentrations in the content samples of feces were determined by gas chromatography (GC) as described previously (Lan et al., 2007). The sample peaks were identified by comparing their retention times with internal standards of acetate, propionate, butyrate, isobutyrate, valerate, isovalerate, and metaphosphoric-crotonic acid.

### Calculation and statistical analyses

All data in the current study were presented as the mean ± SEM. The content of cellulose, and hemicellulose was calculated as follows:

Hemicellulose = NDF – ADF

Cellulose =ADF – (Ash + Lignin)

TDF = IDF + SDF.

Where: the data of acid detergent fiber (ADF) and neutral detergent fiber (NDF) were provided from our previous research (Pu et al., 2020).

The ATTD of all nutrients was calculated as follows:


(1) $$\begin{array}{l} \text{CA}{\text{D}}_{\text{D}}\left(\text{\%}\right)\;=\;100\times \left(1-\left(\text{D}{\text{C}}_{\text{F}}\times \text{AI}{\text{A}}_{\text{D}}\right)/\left(\text{D}{\text{C}}_{\text{D}}\times \text{AI}{\text{A}}_{\text{F}}\right)\right) \end{array}$$

Where: CAD_D_ is the ATTD of nutrients in experimental diets; DC_F_ is the nutrient content in feces; AIA_D_ is the acid insoluble ash in feeds; DC_D_ is the nutrient content in feeds, and the AIA_F_ is the acid insoluble ash content in feces. The average intake of TDF, SDF, and IDF was calculated referring to our previous study (Pu et al., 2020).

Polynomial contrasts were conducted to determine the effects of the increasing dietary fiber level on growth performance, the ATTD of dietary fiber, the activity of fiber degrading enzymes, SCFAs, and microbiota in pigs using SPSS 20.0. For comparing the data of microbial relative abundance, Kruskal–Wallis test was employed, and Mann–Whitney U test was performed for pairwise comparisons. Here, the PICRUSt analysis was used to predict the functions of bacterial communities based on the 16S rRNA gene data (Langille et al., 2013). The factors shaping the dynamics of the swine gut microbiota were disclosed using permutational multivariate analysis of variance (PERMANOVA). The correlations analysis was performed by Spearman's correlation analysis (two-tailed test) using Origin 9.5. Statistical significance was defined as *p* < 0.05. The correlation network analysis of microbiota, growth performance, and average fiber intake was conducted using Cytoscape 3.8.2 (Shannon et al., 2003).

### Data deposition

All the raw data of 16S rRNA amplicon sequencing in this study have been deposited in NCBI's Short Read Archive under the accession number PRJNA726036.

## Results

### Growth performance, average fiber intake, and fiber apparent digestion of finishing pigs

On day 14 of feeding, the body weight, ADG, ADFI, and F/G of finishing pigs was not significantly affected as dietary fiber level increased. Average daily intake of IDF, SDF, and TDF changed quadratically as dietary fiber level increased (*p* < 0.01), and reached the highest in treatment II (Table 1). Meanwhile, average intake of SDF increased linearly as dietary fiber level increased (*p* < 0.01), an average intake of IDF (*p* = 0.069), TDF (*p* = 0.064) had increasing trends (Table 1).

**Table 1:** The effects of short-term feeding of defatted rice bran-containing diets on growth performance in finishing pigs.

**Items**	**Groups**					**SEM**	***p-*** **value**	
	**Control**	**I**	**II**	**III**	**IV**		**Linear**	**Quadratic**
ADFI, kg/d	2.23	2.04	2.09	2.03	2.18	0.06	0.863	0.343
ADG, g/day	457.14	378.57	426.53	400.00	382.86	37.36	0.551	0.891
Bodyweight of day 14, kg	76.20	74.30	75.17	74.63	77.72	2.85	0.633	0.347
F/G	4.90	5.82	5.21	5.29	5.78	0.20	0.454	0.923
Average fiber intake, g/d
IDF	344.00	365.75	543.62	427.09	411.88	17.13	0.069	0.007
SDF	11.08	11.92	19.07	15.77	15.56	0.66	0.001	0.008
TDF	355.94	377.67	560.84	442.86	427.44	17.67	0.064	0.008

**Figure 1:**
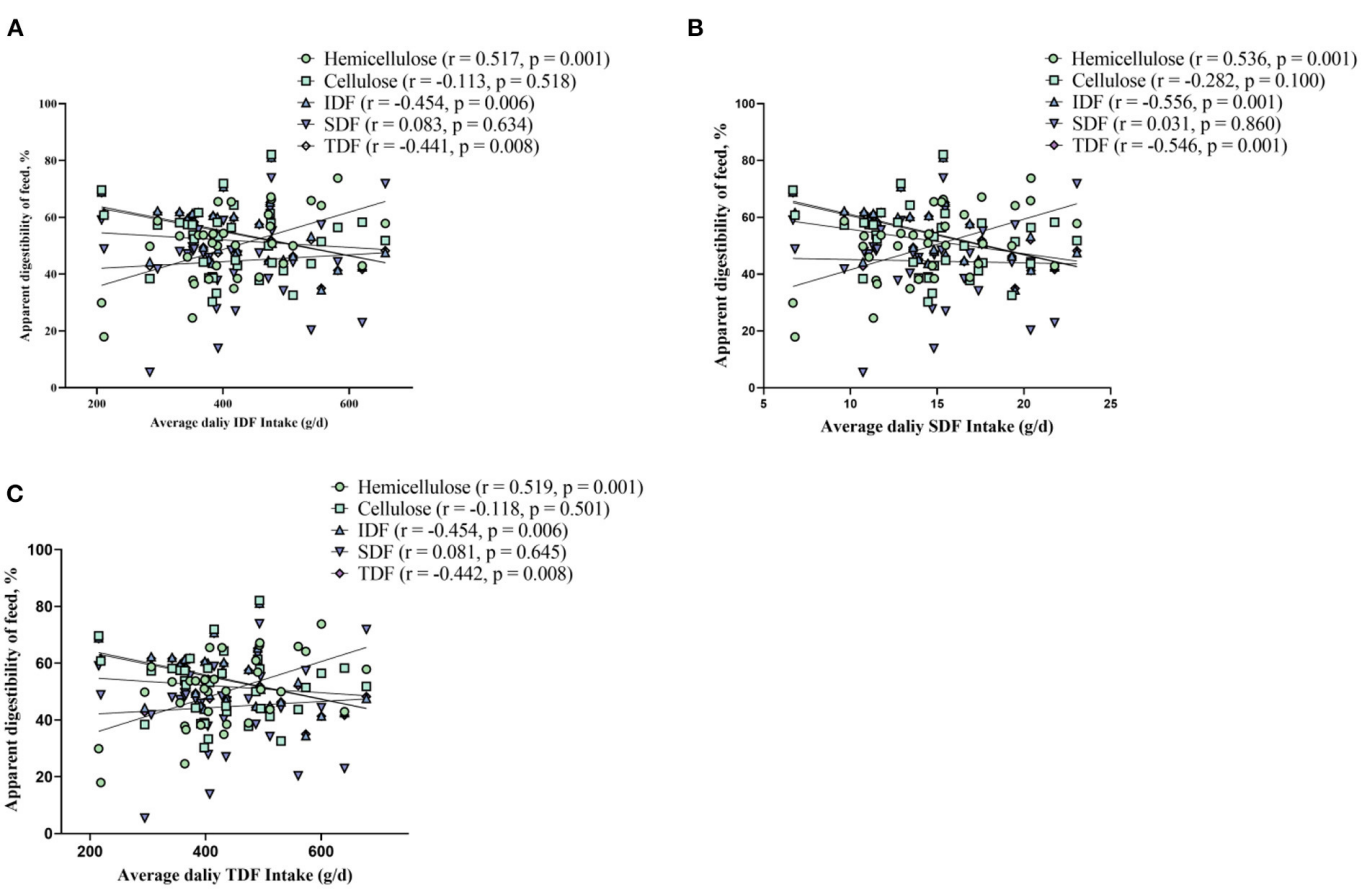
Spearman's correlation analysis between fiber apparent digestibility and average fiber intake of insoluble dietary fiber **(A)**, soluble dietary fiber **(B)**, and total dietary fiber **(C)**, respectively.

The results of ATTD of fiber revealed that ATTD of cellulose, IDF, and TDF decreased (linear, *p* < 0.01), and changed quadratically as dietary fiber levels increased (*p* < 0.01) (Table 2). It should be noted that the effect of fiber on ATTD of cellulose, IDF, and TDF is mild at a lower level (7% DFRB) and accelerated at higher levels (Table 2). ATTD of SDF had a slightly decreased trend (linear, *p* = 0.064). While, ATTD of hemicellulose increased (linear, *p* < 0.05) (Table 2). In addition, through Spearman correlation analysis between average fiber intake and ATTD, we found that with the increase of fiber intake, ATTD of IDF and TDF significantly decreased (*p* < 0.01), while ATTD of hemicellulose significantly increased (*p* < 0.01) (Figure 1).

**Table 2:** The effects of short-term feeding of DFRB-containing diets on apparent total tract digestibility of fiber in finishing pigs.

**Items**	**Groups**					**SEM**	***p-*** **value**	
	**Control**	**I**	**II**	**III**	**IV**		**Linear**	**Quadratic**
Cellulose	66.23	57.57	50.32	46.3	38.59	1.98	<0.000	<0.000
Hemicellulose	37.97	49.61	54.56	52.07	54.20	2.02	0.030	0.028
IDF	59.88	61.24	51.31	50.22	47.98	1.31	<0.000	<0.000
SDF	53.98	49.05	45.98	36.74	45.16	2.11	0.064	0.022
TDF	59.67	61.20	51.45	48.96	47.73	1.34	<0.000	<0.000

### Fecal microbial diversity of suhuai finishing pigs and its relationships with fiber intake

**Figure 2:**
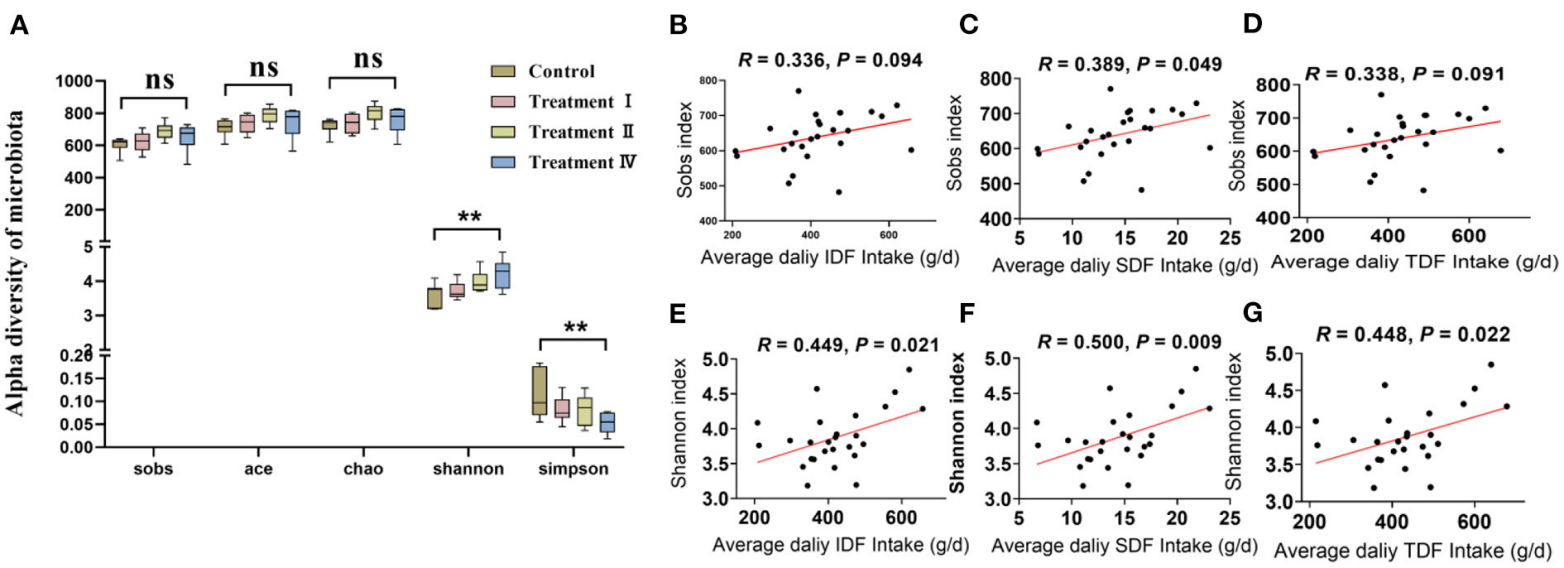
Comparison of microbial community between treatment groups and the control group. Alpha diversity of microbial communities **(A)**. Spearman's correlation between Sobs index with insoluble dietary fiber **(B)**, soluble dietary fiber **(C)** and total dietary fiber **(D)**, respectively. Spearman's correlation between Shannon index with insoluble dietary fiber **(E)**, soluble dietary fiber **(F)**, and total dietary fiber **(G)**, respectively. One-way ANOVA followed by LSD *post-hoc* test was conducted. *p < 0.05, **p < 0.01, ns, not significant.

Firstly, the established rarefaction curves at the OTU level showed that extra sampling would be of limited benefit (Supplementary Figure S1). One-way ANOVA analysis with followed by LSD *post*-*hoc* test for microbial alpha diversity among groups revealed that fecal microbial diversity (Shannon and Simpson) of pigs in treatment IV was significantly higher than those in the control (*p* < 0.05). While the richness index (Sobs, Ace and Chao) had no change with the increasing in dietary fiber level (Figure 2A). In addition, there was a significant positive correlation between average fiber intake (IDF, SDF, and TDF) and intestinal microbial diversity (Shannon, *p* < 0.05) (Figures 2E–G). A weak positive correlation between average fiber intake (IDF, SDF and TDF) and intestinal microbial richness (Sobs) was also observed (*p* < 0.10) (Figures 2B–D). We further performed PERMANOVA to test which nutrient intake contributed most to the gut microbiome of the pig. The results revealed that average intake of various fibers was dominant, which explained the majority of the microbial variation, while average intake of crude protein (CP) and ether extract (EE) had mild effects on microbial variation (Supplementary Table S3).

### Dynamic change of microbial composition and functions as dietary fiber level increased

**Figure 3:**
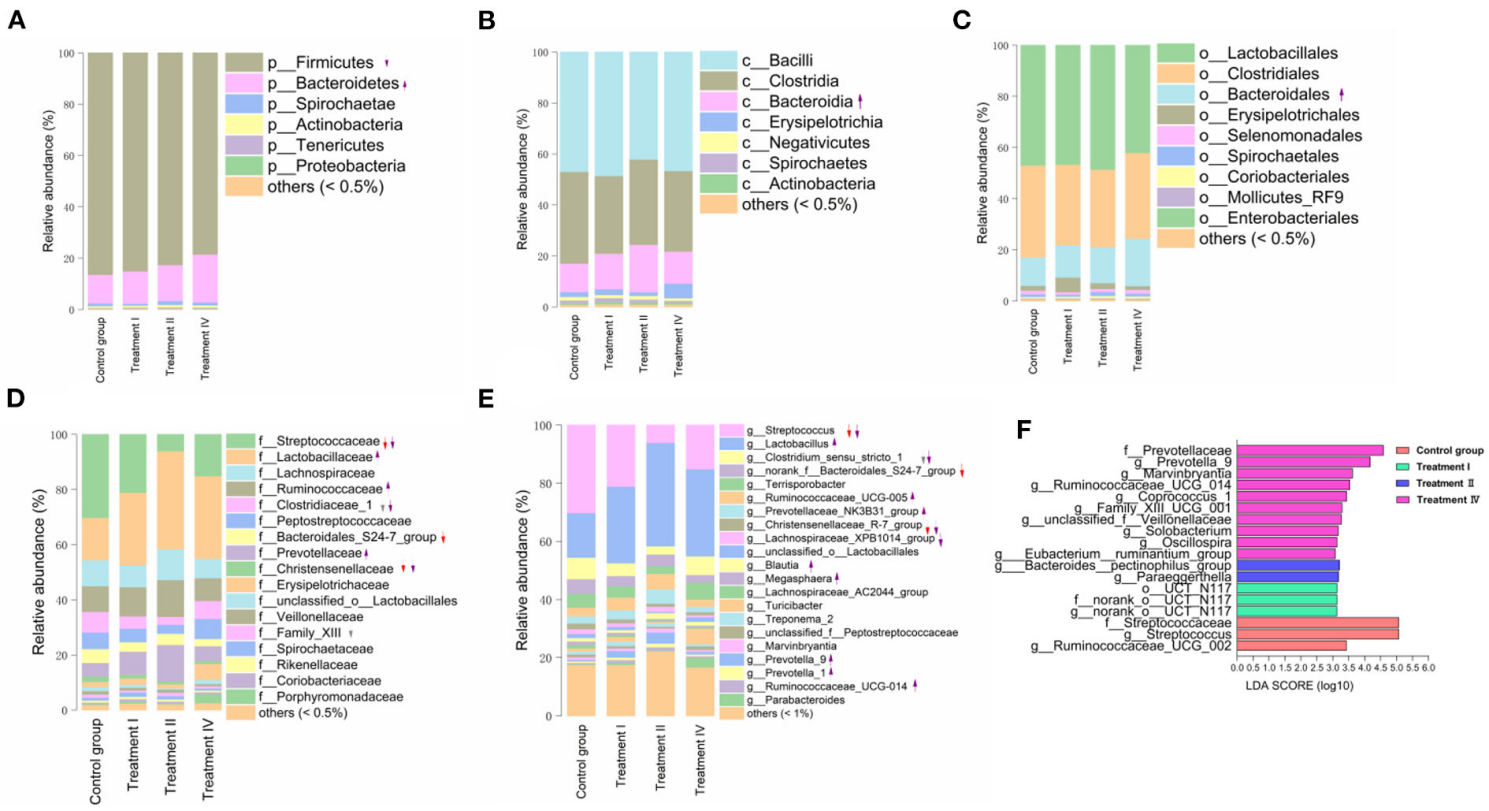
Shifts in fecal microbial composition of pigs at phylum **(A)**, class **(B)**, order **(C)**, family **(D)**, and genus **(E)** levels as the increase of dietary fiber. The LEfSe analysis of intestinal bacterial communities **(F)**. Upward and downward arrows with solid line in **(A–E)** mean that the proportions of the corresponding microbial taxa significantly increased or decreased, compared with those in the control group, respectively (*p* < 0.05). The arrows with dotted line in **(A–E)** mean that the relative abundance of the corresponding microbial taxa has increased or decreased trend, respectively (*p* < 0.10). Red arrows mean comparison of treatment I vs. the control group. Gray arrows mean a comparison of treatment II vs. the control group. Purple arrows mean comparison of treatment IV vs. the control group. Letters p, c, o, f and g at the head of the names of taxon represent phylum, class, order, family and genus, respectively.

The microbial composition (from phylum to genus) of four groups is shown in Figures 3A–E. Briefly, at the phylum level, Firmicutes (83.12%), Bacteroidetes (14.09%), and Spirochaetae (1.17%) were the top three dominant phyla in all pigs (Figure 3A). In genus, *Lactobacillus* (26.81%), *Streptococcus* (18.09%), *Clostridium_sensu_stricto_1* (5.27%), *Terrisporobacter* (4.27%) and *Ruminococcaceae_UCG-005* (3.82%) were the top five dominant genera in all samples (Figure 3E).

**Figure 4:**
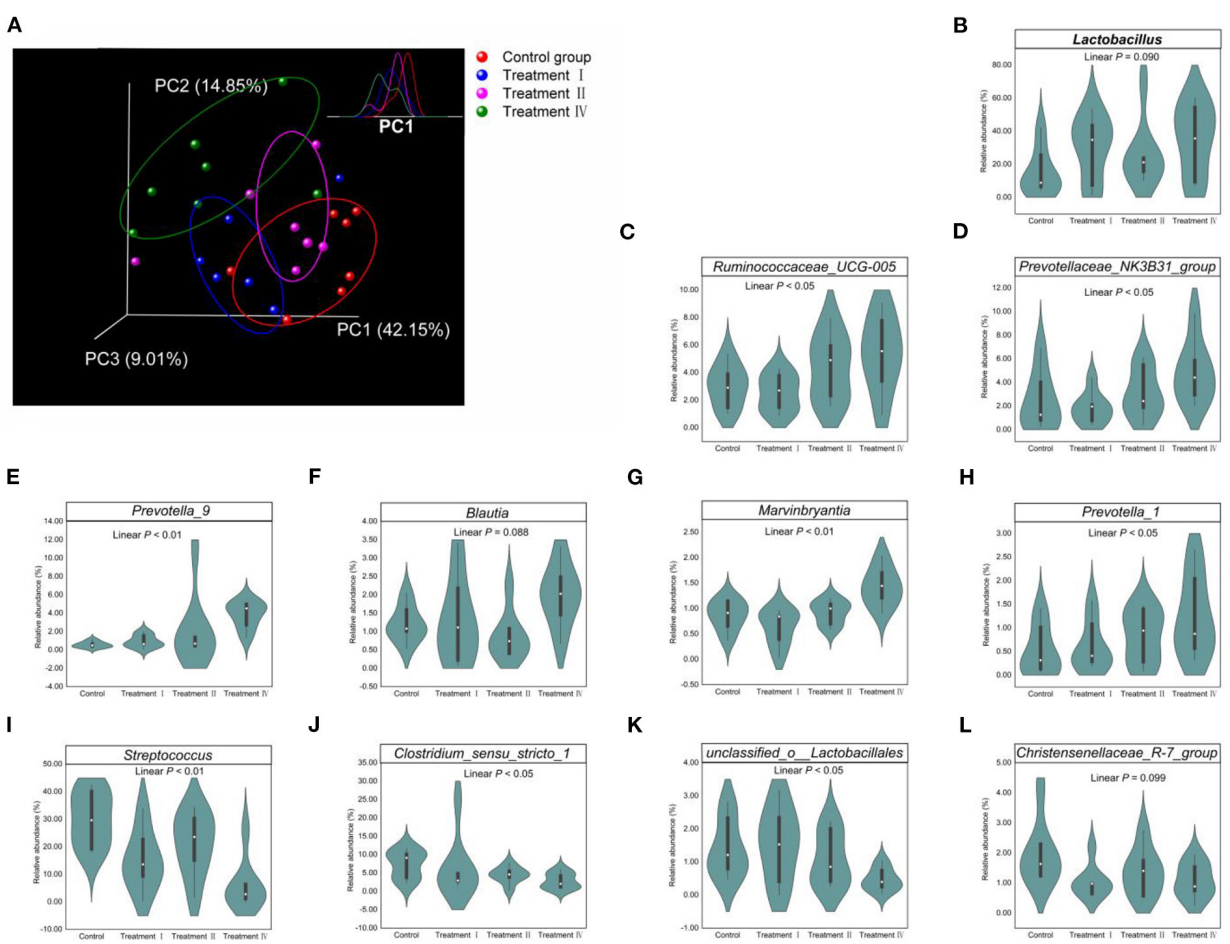
Effect of dietary fiber on fecal microbiota. **(A)** Scatterplot from PCoA of fecal bacterial communities based on Bray_Curtis distance. Top right panel showed that the density distribution of the projection in PC1. **(B–L)** Linear change of microbial relative abundance in the top 21 genera. Only change with statistical support (*p* < 0.10) was showed in this picture. Polynomial contrasts analysis was conducted.

**Figure 5:**
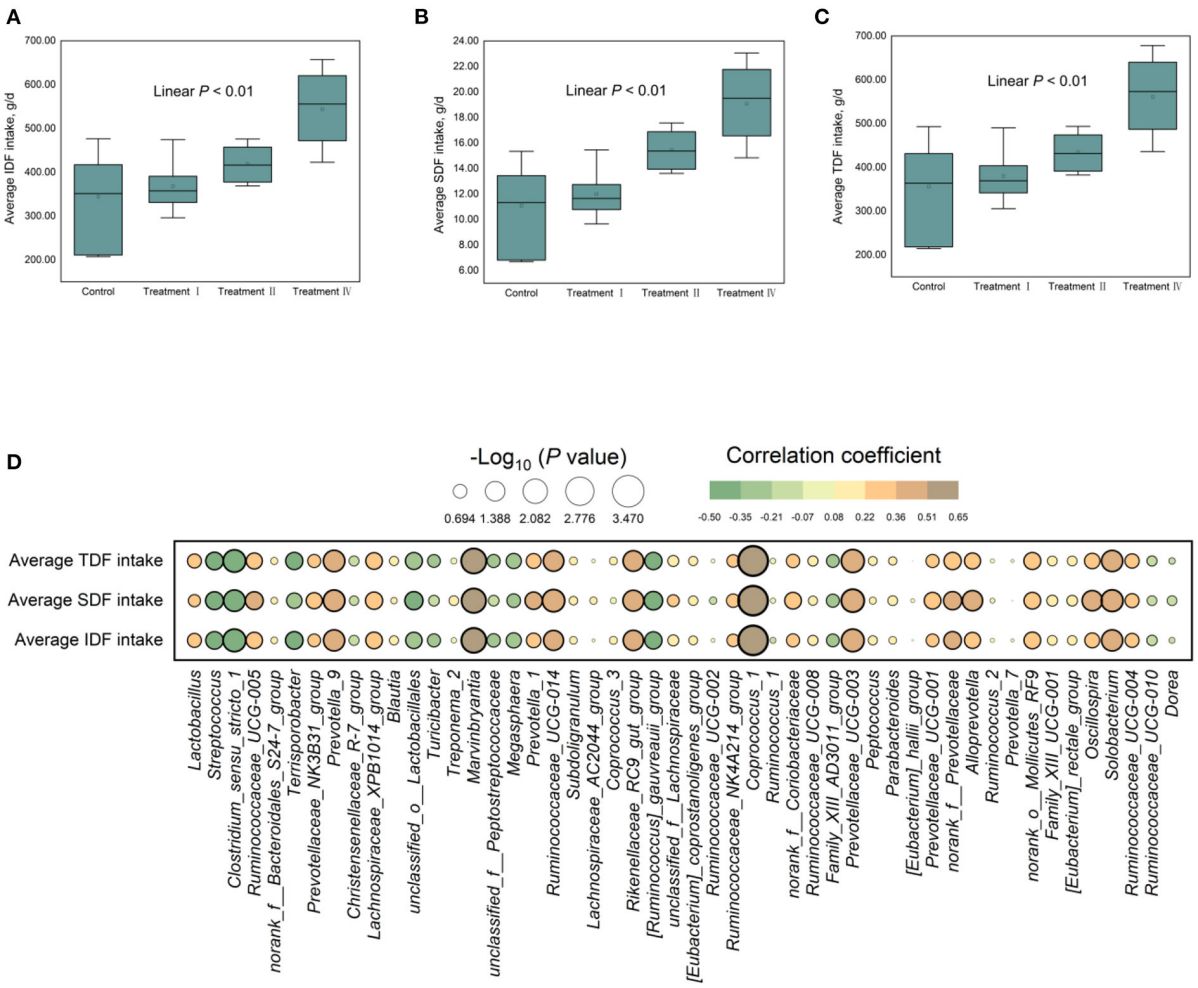
**(A–C)** The dietary fiber intake among four groups and **(D)** their correlation analysis with microbiota (Top 50).

Changes in fecal microbial composition at different taxonomic levels (Figures 3A–E) between each of the treatment groups and the control were measured by a Mann–Whitney U test. Notably, at the phylum level (relative abundance was more than 0.5%), the proportion of Bacteroidetes in treatment IV was higher than that in the control (*p* < 0.05) (Figure 3A), and the relative abundance of Firmicutes had a decreased trend (*p* = 0.061). In the top 21 genera (relative abundance was more than 1%), we found that the main differences occurred between treatment IV and the control group (Figure 3E). Of them, the relative abundance of *Prevotella_9* (*P* < 0.01), *Marvinbryantia* (*p* < 0.05), and *Blautia* (*p* < 0.05) in treatment IV was higher than that of in the control. And the relative abundance of *Ruminococcaceae_UCG-005, Lactobacillus, Prevotellaceae_NK3B31_group*, and *Prevotella_1* in treatment IV had an increasing trend when compared to the control group (*p* < 0.10) (Figure 3E). While the relative abundance of *Streptococcus* in treatments I and IV, and the relative abundance of *Clostridium_sensu_stricto_1* in treatment IV were lower than those in the control group, respectively (*p* < 0.05) (Figure 3E). Linear discriminant analysis effect size (LEfSe) confirmed the above results (Figure 3F). Principal coordinate analysis (PCoA) based on a Bray_Curtis distance metric highlighted a clear shift of fecal microbial community from the control group to treatment IV. In particular, there was a significant separation between treatment IV and the control group (Adonis, *p* < 0.05) (Figure 4A). To follow up the changes of intestinal microbiota with dietary fiber level, the data of the above microbiota were used to contrast statements analysis. The results revealed that the relative abundance of *Lactobacillus* (*p* = 0.090), *Ruminococcaceae_UCG-005* (*p* < 0.05), *Prevotellaceae_NK3B31_group* (*p* < 0.05), *Prevotella_9* (*p* < 0.01), *Blautia* (*p* = 0.088), *Marvinbryantia* (*p* < 0.01) and *Prevotella_1* (*p* < 0.05) increased linearly with the increasing of dietary fiber level (Figures 4B–H). While the relative abundance of *Streptococcus* (*p* < 0.01), *Clostridium_sensu_stricto_1* (*p* < 0.05) and *unclassified_o_Lactobacillales* (*p* < 0.05) decreased linearly (Figures 4I–L). With the increasing of DFRB level, the dietary fiber intake of pigs increased (Figures 5A–C). Moreover, the correlation analysis between microbiota (Top 50) and dietary fiber intake revealed that dietary fiber intake was a dominant factor affecting the microbial composition (Figure 5D).

**Figure 6:**
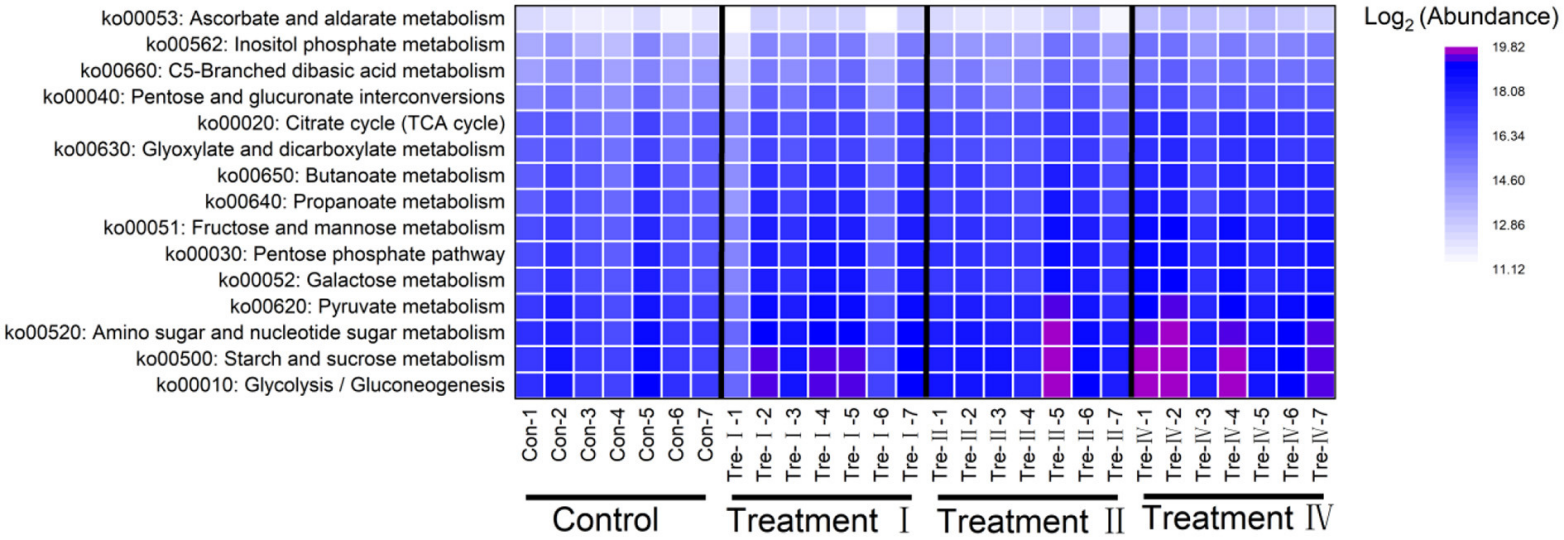
Distribution of carbohydrate metabolism related pathways of fecal microbiota.

Subsequently, PICRUSt analysis was performed to investigate the effects of dietary fiber on microbial functions. Dietary fiber belongs to carbohydrate, thus KEGG pathways related to carbohydrate metabolism got noticed. Glycolysis/Gluconeogenesis, Starch and sucrose metabolism, Amino sugar and nucleotide sugar metabolism, Pyruvate metabolism were main carbohydrate metabolism-related pathways (Figure 6). Notably, abundance of most carbohydrate metabolism related pathways increased with the increase of dietary fiber level (Figure 6). In addition, the activity of fiber-degrading enzymes analysis revealed that activity of carboxymethyl cellulase and microcrystalline cellulase significantly increased with the increasing of dietary fiber level (linear, *p* < 0.05) (Supplementary Table S4).

### Fiber-degrading potential of microbiota in pigs

**Figure 7:**
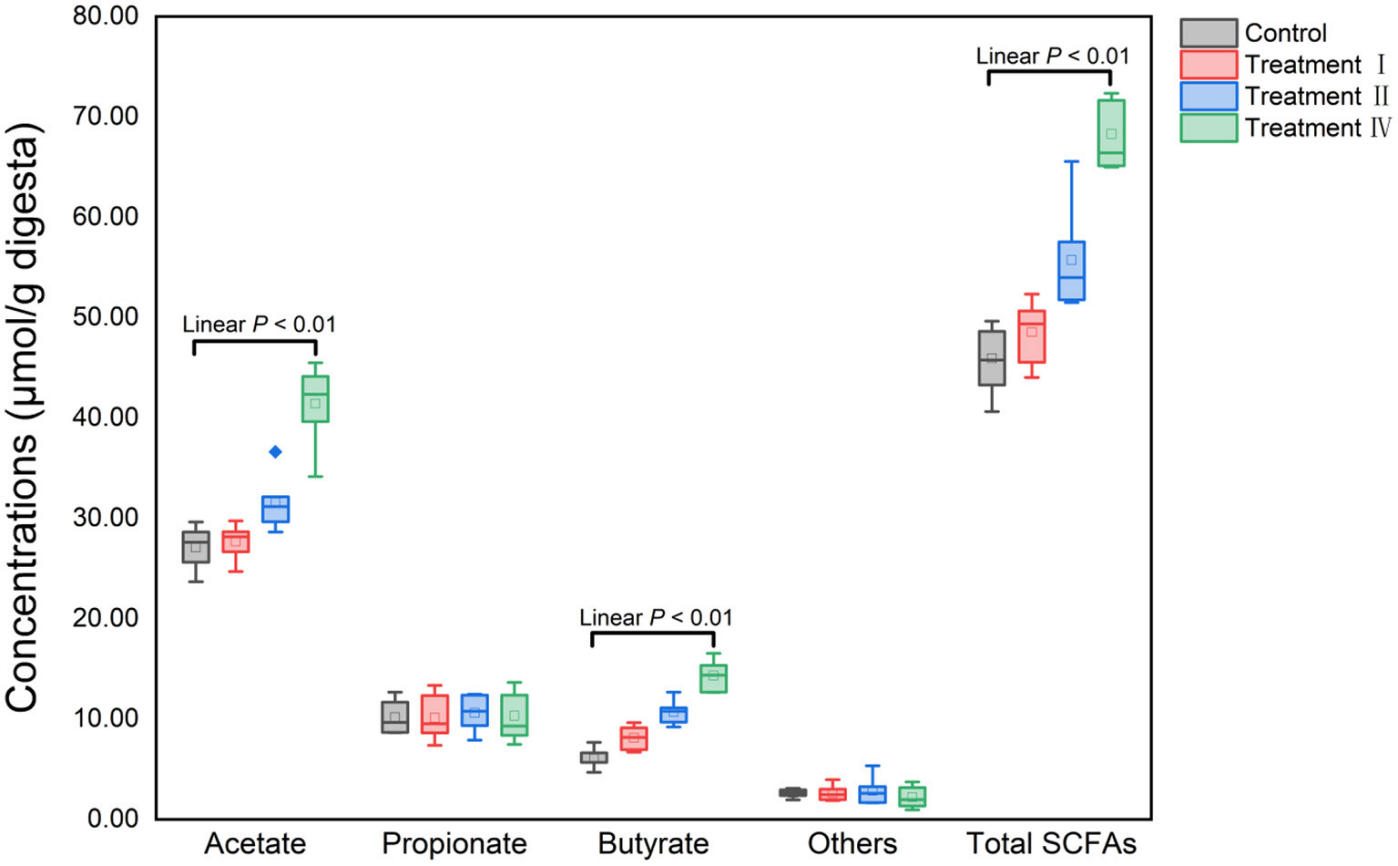
Variation of fecal SCFAs concentrations among four groups with the increasing of dietary fiber level. Others: isobutyrate, valerate and isovalerate.

**Figure 8:**
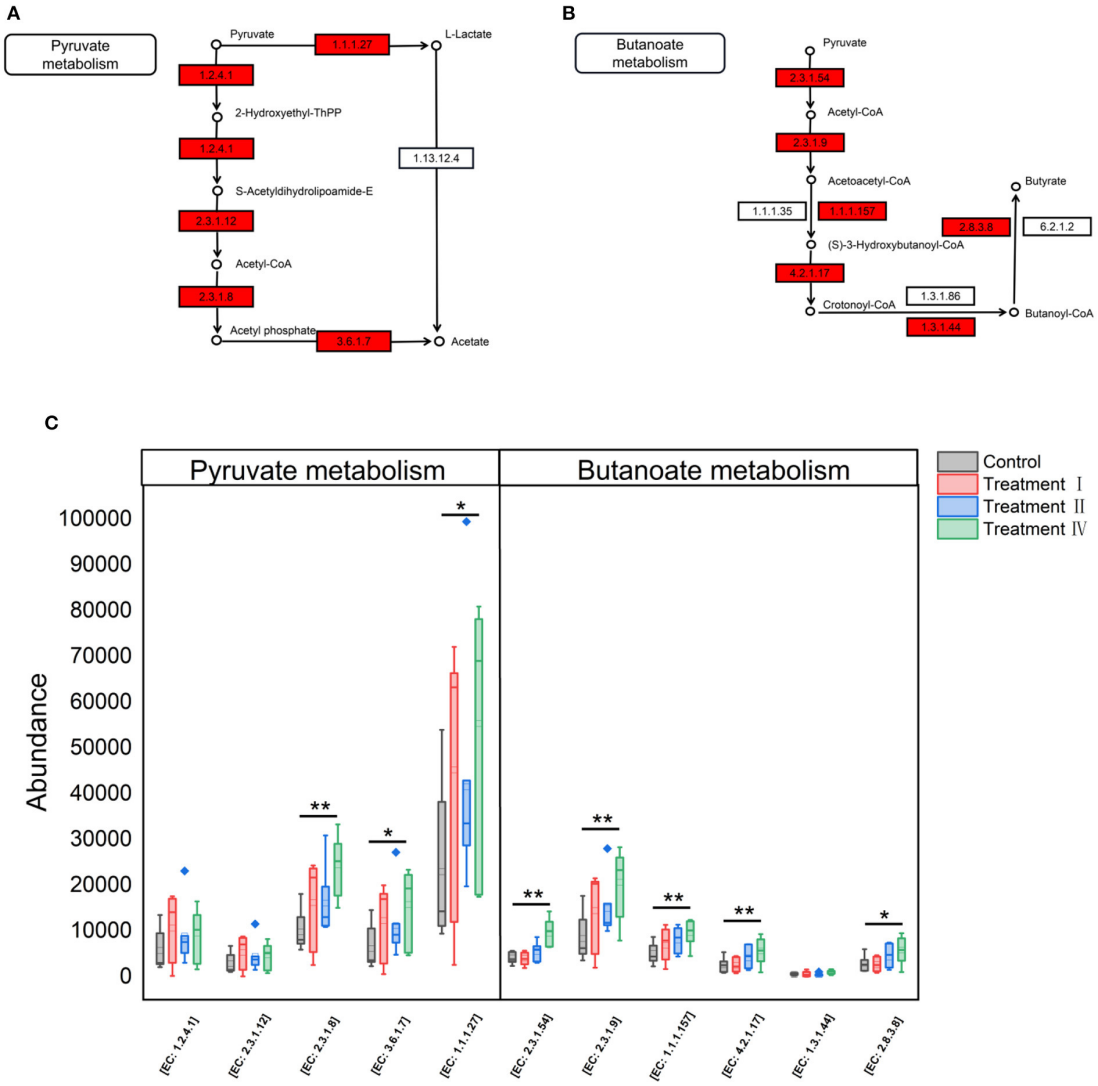
KEGG pathways and enzymes of microbiota related to fiber degradation. **(A)** Enzymes related to acetate production in pyruvate metabolism. **(B)** Enzymes related to butyrate production in butanoate metabolism. The enzyme with red filling color is predicted by the special gene set in this study. **(C)** Variation of enzymes among four groups with the increasing of dietary fiber level. ^*^P < 0.05, ^**^P < 0.01.

Fecal SCFAs concentrations were determined to assess dietary fiber fermentation potential of intestinal microbiota. Concentrations of acetate, butyrate and total SCFAs significantly increased with increasing of dietary fiber level (Figure 7). To investigate which microbe might affect the yield of acetate, butyrate. 16S genes of different microbes (11 genera: Figures 4B–L) were constructed into a special gene set. Then, the enzymes of the special gene set involved in pyruvate metabolism (ko00620), butanoate metabolism (ko00650) were further investigated. Five kinds of enzymes related to acetate production were enriched in pyruvate metabolism (Figure 8A), and six kinds of enzymes related to butyrate production in butanoate metabolism (Figure 8B). Among these enzymes, the abundance of Phosphate acetyltransferase [EC: 2.3.1.8], Acylphosphatase [EC: 3.6.1.7], L-lactate dehydrogenase [EC: 1.1.1.27], Formate C-acetyltransferase [EC: 2.3.1.54], Acetyl-CoA C-acetyltransferase [EC: 2.3.1.9], 3-hydroxybutyryl-CoA dehydrogenase [EC: 1.1.1.157], Enoyl-CoA hydratase [EC: 4.2.1.17] and Acetate CoA-transferase [EC: 2.8.3.8] increased with the increasing of dietary fiber level (linear, *P* < 0.05) (Figure 8C).

### Potential core members of fecal microbiome related to fiber utilization and growth performance in finishing pigs

**Figure 9:**
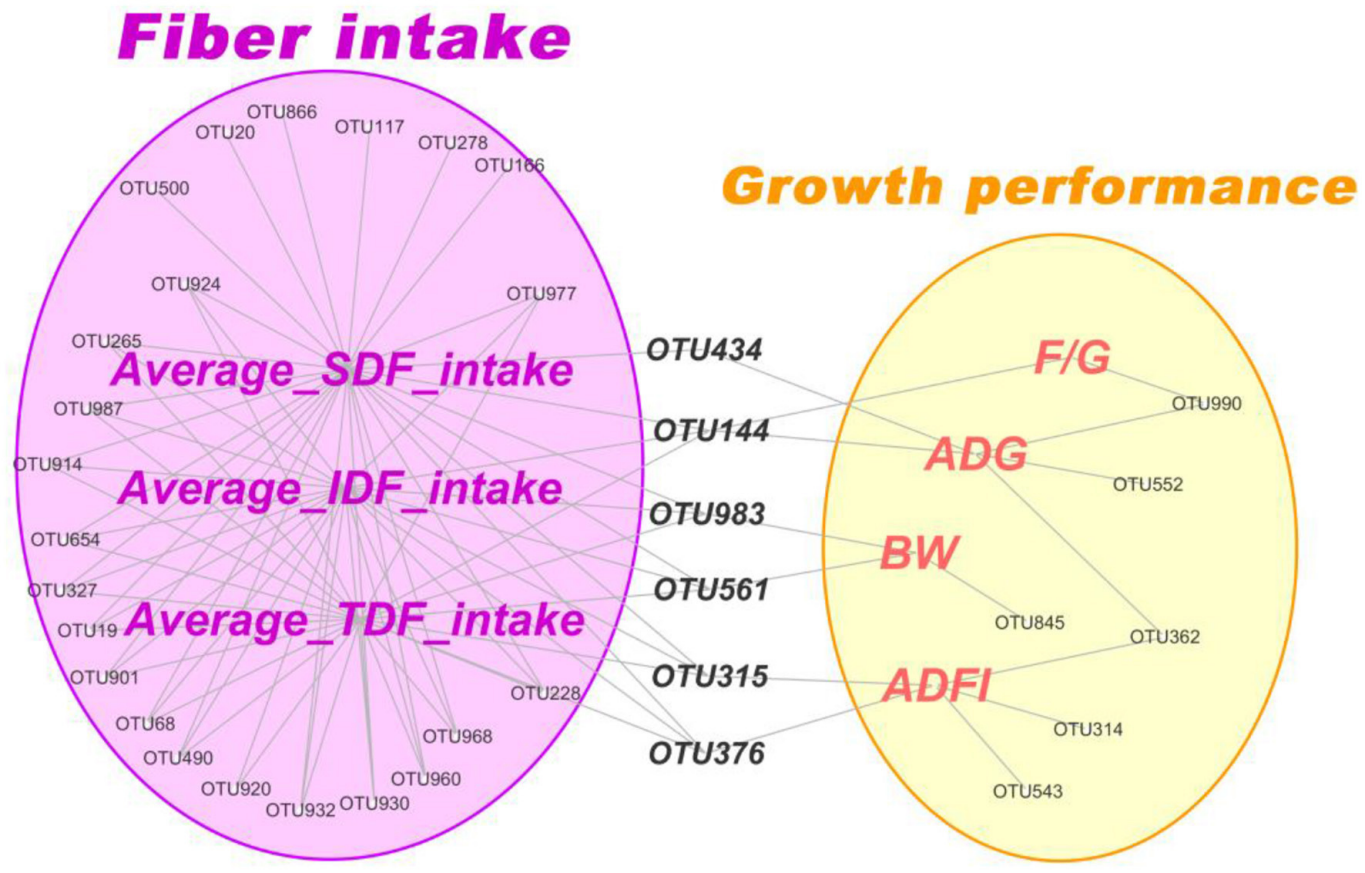
Correlation network analysis between dietary fiber intake and growth performance of pigs with dominate microbiota (top 100 OTUs) in feces. Only OTUs significantly related to these indicators (*P* < 0.05) were shown in network.

To further accurately screen microbes closely related to fiber digestion and growth performance of pigs. Firstly, correlation network analysis between the fiber intake of IDF, SDF, TDF and growth performance of pigs with main microbiota (top 100 OTUs) was conducted, respectively. The result revealed that 15 OTUs, 21 OTUs and 15 OTUs positively correlated with the average intake of TDF, IDF, and SDF, respectively (Spearman correlations > 0.40, *P* < 0.05) (Figure 9). Then, these OTUs were mainly annotated to family Prevotellaceae, Ruminococcaceae and Lachnospiraceae by seeking the Silva (SSU123) 16S rRNA database. While there were only 5 OTUs, 7 OTUs and 7 OTUs, annotated to genus *Streptococcus*, significantly negatively correlated with the average intake of TDF, IDF and SDF, respectively (Spearman correlations < -0.38, *P* < 0.05) (Figure 9). In addition, there were just a few OTUs significantly correlated with growth performance of pigs (including F/G, ADG, BW and ADFI) (*P* < 0.05). It is remarkable that OTU434, OTU144, OTU983, OTU561, OTU315, and OTU376 simultaneously significantly correlated with fiber intake and growth performance of pigs. After these 6 OTUs were annotated against the Silva (SSU123) 16S rRNA database, we found that the 6 OTUs were the members of genus *Oscillospira, Solobacterium, Clostridium_sensu_stricto_1, Prevotellaceae_UCG-003, Ruminococcaceae_UCG-008* and *Marvinbryantia*, respectively.

## Discussion

### Effects of short-term high fiber feeding on growth performance and ATTD of fiber in finishing pigs

The inclusion of dietary fiber in pig feeding is often limited because they can have anti-nutritive properties, including some suppressors in the digestibility of dietary protein and energy (Noblet and Le Goff, 2001). A large number of studies reported that the effects of high fiber diet on growth and nutrient digestion of pigs. Casas et al. (2018) used basal diet, 10, 20, and 30% full fat rice bran to feed growing-finishing pigs respectively for 35 days, and found ADFI decreased linearly and F/G decreased linearly. Lyu et al. (2018) fed growing pigs with 220 g/kg sugar beet pulp and 200 g/kg soybean hulls, respectively, and concluded that high fiber diets had a negative effect on nutrients digestibility and energy values. While Wang et al. (2018) fed the pigs with the diets containing 5, 10, and 15% alfalfa meal for a 28-day experimental period and found that ingestion of alfalfa meal-contained diets significantly increased feed utilization efficiency, however, the ADFI and ADG of pigs were no affected. It is worth noting that most studies are based on a 28-day or longer feeding phase. There are few studies have evaluated the response of pigs' growth performance to high-fiber diet in a short-term feeding. Our present study demonstrated the growth performance of pigs was not affected by high fiber diet after a 14-day feeding period. It should be noted that as the dietary fiber level increased, the amount of apparent fiber digestion of Suhuai pigs increased in the current study. It was estimated that microbial fermentation products (Short chain fatty acids, SCFAs) contribute 15 % for energy requirement of growing-finishing pigs (Dierick et al., 1989). Therefore, we speculated that the increased amount of fiber digestion of Suhuai pigs in short-term high fiber feeding, which might promote the metabolic activities of microbiota, thereby products adequate SCFAs for energy requirement of Suhuai pigs. Notably, the calculated values of CP, ME and standardized ileal digestible (SID) amino acids were slightly different among groups, which may affect the growth and digestion of pigs. In the present study, the main difference among five diets was fiber, which was mainly caused by different DFRB addition. However, in order to make protein, energy, amino acids, calcium and phosphorus of five groups nearly the same, we also slightly adjusted the proportions of soybean meal and wheat bran in each group. Because there was also fiber in soybean meal and wheat bran, the difference of total fiber in each diet may also be related to the different proportions of soybean meal and wheat bran, which is a limitation of our study. In addition, the physical composition and chemical properties of different feed materials (such as corn and DFRB) may also affect the digestion of diets for pigs. Therefore, in this study, even if the nutritional levels of the five feed formulations are almost the same, it is still necessary to understand the influence of the properties of raw materials. And in the future research, there is a need that eliminating or weakening the interference of these factors should be more strict.

Subsequently, our current study and a previous study (Pu et al., 2019) revealed that the digestibility of protein, energy, and various insoluble fibrous ingredient strikingly decreased with the increase in dietary fiber level, which was consistent with observations by Wilfart et al. (2007b) and Urriola and Stein (2009). Clearly, the inclusion of dietary fiber in pig diets is sometimes limited because it can have anti-nutritive properties. These include a reduction in the digestibility of dietary energy and protein (Noblet and Le Goff, 2001) which may lead to an inadequate amino acid, particularly threonine, absorption (Blank et al., 2012). Although fibrous dietary constituents can have higher crude protein levels than non-fibrous constituents, on average, approximately 30% of nitrogen is bound to neutral detergent fiber, which is not available to the animal (Bindelle et al., 2008). In this current study, the ATTD of cellulose, IDF, and TDF of Suhuai finishing pigs was not affected when the TDF level reached 17.75% (as feed basis), which was slightly different from the result of previous data. These results suggested that pigs were undergoing a process of adaption to high fiber diets, and this adaptability was further reflected with the extension of feeding time. When came to day 28 of the experimental period, Suhuai finishing pigs could tolerate the diet containing 19.10% TDF (Pu et al., 2020). We speculated that the stable and increasing adaptability to the high dietary fiber of Suhuai finishing pigs might be related to efficient fiber degradation by distal gut microbiota. Interestingly, the ATTD of hemicellulose increased with the fiber level, which was consistent with the finding after feeding of 28 days (Pu et al., 2020). These findings indicated that soluble fiber ingredients were easier to digested by Suhuai pig than insoluble fiber ingredients, which was consistent with observations by Noblet and Le Goff (2001).

It was unexpected but needs to be noted that the ATTD of SDF seemed to be lower than of IDF (no statistical test), which is consistent with the findings of Navarro et al. (2019). Bindelle et al. (2008) found too high a content of insoluble fiber in diet would interfere with the contact opportunities of other nutrients and digestive enzymes (Bindelle et al., 2008). In the current study, the fiber in DFRB was almost all insoluble (Supplementary Table S1). Therefore, we speculated that the low digestibility of SDF might be due to the less chance of contact with fiber-degrading enzyme or the contact area being limited. On the contrary, the IDF in DFRB had more contact opportunities and contact areas of IDF, thus it had higher digestibility.

### Effects of short-term high fiber feeding on fecal microbial composition and microbial functions

Generally, it has been well demonstrated that dietary fiber plays a positive role in maintaining the diversity of the gut microbial community and intestinal health of pigs (Knudsen, 2001; Jha et al., 2010). A high-fiber diet could also increase the activity of fiber-degrading related bacteria in the large intestine of growing pigs (Varel, 1987; Metzler and Mosenthin, 2008). These conclusions were reconfirmed by our current study, we found pigs in treatment IV (24.11% TDF) had higher proportions of several fiber-degrading related bacteria genera in feces. For instance, three members of the family Prevotellaceae, were reported that could utilize arabinoxylans (Pieper et al., 2015). *Marvinbryantia*, a member of Lachnospiraceae, is a kind of cellulose and methylcellulose degradation bacteria (Abdessamad et al., 2013). Ruminococcaceae and Lachnospiraceae were also reported can produce enzymes that degrade carbohydrates (Abdessamad et al., 2013). In addition, *Blautia* is an anaerobic Gram-positive bacterium found in the intestine (Lawson and Finegold, 2015). In general, members of the genus *blautia* were reported to produce acetic acid, ethanol, hydrogen, lactic acid or succinate as the final product of glucose fermentation (Liu et al., 2008; Seong-Kyu et al., 2013). At the same time, we also found that some fiber-degrading related bacteria changed in the opposite way, such as *Streptococcus* (Hutkins and Morris, 1987) and *Clostridium_sensu_stricto_1* (Zhu et al., 2011). Remarkably, our study showed the fecal microbial abundance (Chao and Sobs index) and several genera of Ruminocacae and Lachnospiraceae of Suhuai finishing pigs in treatment IV (the diet containing 24.11% TDF) were significantly higher than those in the control group. Interestingly, these beneficial changes in treatment IV transferred to treatment II (the diet containing 19.10% TDF) after feeding of 28 days (Pu et al., 2020). We speculated that it might be due to the change of intestinal microbiota community caused by short-term intake of high fiber diet (containing 24.11%), specifically the increasing in the abundance of dominant fiber-degrading bacteria, so as to digest more fiber to provide energy for pigs. However, with the extension of the experimental period, intestinal tract of pigs could not adapt the high-fiber diet (24.11% TDF), intestinal microbiota community returned to the original level.

Conversions of dietary fiber to monosaccharides in the intestinal tract involved a number of reactions mediated by the microbial enzymatic repertoire, and the majority of end products from fermentation of dietary fiber are SCFAs (Koh et al., 2016). In the current study, we found that SCFAs production and microbial pyruvate metabolism, and butanoate metabolism increased with increasing dietary fiber. Moreover, the activity of carboxymethyl cellulase and microcrystalline cellulase also increased. Therefore, we speculated that increasing dietary fiber might promote the decomposition of complex fiber structures into oligosaccharides, the metabolism of carbohydrates and energy, and other microbial functions, which thereby resulted in the linear increasing of average fiber digestion. These results are consistent with our previous study (Pu et al., 2020). In addition, it is widely agreed that SCFAs, the end production of fiber fermentation by large gut microbiota, contribute to the animal energy supply and regulate the growth of gut epithelial cells. Dierick et al. (1989) estimated that fermentation products of dietary fiber contribute about 0.15 for growing-finishing pigs and about 0.3 for gestating sows (Varel and Yen, 1997). Thus, we speculated that the reason for the unchanged growth performance of Suhuai pigs in this experiment may be related to the enhancement of microbial fiber-degrading functions and the increase of microbial metabolites such as SCFAs.

### Potential core members of gut microbiota related to fiber utilization and growth performance of finish pigs

Some larger scale studies have remarkably expanded our understanding of the swine gut microbiome (Chen et al., 2017; Han et al., 2018). Recent studies have also filled some knowledge gaps of the swine gut microbiota, with respect to digestibility (He et al., 2018), and growth performance (Tsai et al., 2018). In this current study, we identified numerous OTUs, that positively correlated with fiber intake, and were mainly annotated to the family Prevotellaceae, Ruminococcaceae, and Lachnospiraceae. These bacterial families were linked to the fermentation of plant-derived non-starch polysaccharides to SCFAs (El Kaoutari et al., 2013; Rajilic-Stojanovic and de Vos, 2014). Simultaneously, these bacterial taxonomies were also related closely to growth performance (E.G., ADG, ADFI, F/G, and BW). These results are consistent with observations by Wang et al. (2019b). In addition, the relative abundance of the members of the genus *Streptococcus* strikingly decreased with the increase of dietary fiber, which is consistent with observations by Zhang L. et al. (2016); Zhang Y. Q. et al. (2016). Although the specific species *Streptococcus thermophilus* was reported to have the function of metabolizing the monosaccharide fructose (Hutkins and Morris, 1987), the *Streptococcus* spp. were associated with the development of multiple metabolic disorders (Zeng et al., 2016). The above results suggested that the increase of dietary fiber could potentially promote the increase of probiotics' abundance, and inhibit the growth of potentially pathogenic bacteria, which improved the intestinal health of finishing pigs.

The difference in the relative abundance of several fiber-degrading related bacterial genera in this study were the dominant donors to distinguish microbial compositions among groups (especially between treatment IV and control group). Microbial diversity analysis and PCoA analysis demonstrated that a 14-day high-fiber (24.11% TDF) diet promoted the change in the fecal microbiota community. In addition, it was worth noting that the characteristics of fecal microbiota in treatment I and II were almost the same as those in the control group, and the proportion of their main bacterial categories (genus level, relative abundance ≥1%) were nearly the same. We speculated that the distal gut microbial community of Suhuai finishing pigs was not affected by the diet containing 17.75% TDF, and could digest enough fiber to maintain the ATTD of dietary fiber. While when TDF continued to reach 24.11%, the significant increase in dietary fiber intake promoted the relative abundance of fiber-degrading related microbiota (a part of members of the family Prevotellaceae, Ruminococcaceae, and Lachnospiraceae) and strengthened fiber-degrading related microbial functions. However, even if more fiber was digested, it still could not prevent decreasing in overall fiber digestibility.

## Conclusion

The present study revealed that the growth performance of finishing pigs was not affected by short-term high fiber feeding, but the ATTD of insoluble fiber strikingly decreased with increasing dietary fiber level during a 14-day feeding period. From the perspective of the ATTD of fiber, Suhuai finishing pigs adapted to the diet containing 17.75% TDF after a 14-day feeding period, and this adaptability was further reflected with the extension of feeding time. In addition, after a 14-day feeding period, the increase in dietary fiber level induced the increase of the members of the family Prevotellaceae, Ruminococcaceae, and Lachnospiraceae, increasing of SCFAs production, and enhancing the microbial pyruvate metabolism and butanoate metabolism.

## Data availability statement

The datasets presented in this study can be found in online repositories. The names of the repository/repositories and accession number(s) can be found in the article/Supplementary material.

## Ethics statement

The animal study was reviewed and approved by Institutional Animal Welfare and Ethics Committee of Nanjing Agricultural University, Nanjing, China. Written informed consent was obtained from the owners for the participation of their animals in this study.

## Author contributions

RH and PL: conceptualization, methodology, software, and writing—review and editing. GP: data curation and writing—original draft preparation. TD, BW, HL, and KL: formal analysis. LH, WZ, and PN: validation and supervision. All authors contributed to the article and approved the submitted version.

## Funding

This work was supported by the National Natural Science Foundation (31872318), the Key Project for Jiangsu Agricultural New Variety Innovation (PZCZ201732), the Funding of Independent Innovation of Agricultural Science and Technology of Jiangsu Province (CX (20) 1003), the project of Jiangsu Agricultural (pig) Industry Technology System [JATS (2020) 179, JATS (2020) 399], the project of Huaian Science and Technology Planning (HAN201901), and the Science and Technology Special Project of Jiangsu Subei (SZ-HA2019016).

## Conflict of interest

The authors declare that the research was conducted in the absence of any commercial or financial relationships that could be construed as a potential conflict of interest.

## Publisher's note

All claims expressed in this article are solely those of the authors and do not necessarily represent those of their affiliated organizations, or those of the publisher, the editors and the reviewers. Any product that may be evaluated in this article, or claim that may be made by its manufacturer, is not guaranteed or endorsed by the publisher.
