# Supplementary material for: Effects of short-term feeding with high fiber diets on growth, utilization of dietary fiber, and microbiota in pigs
Source: Front Microbiol. 2022 Jul 27;13:963917. doi: 10.3389/fmicb.2022.963917 (PMC9363921; doi:10.3389/fmicb.2022.963917)
Supplement: Supplementary file 1 [file Data_Sheet_1.docx]

**Table S1** Raw material composition and a part of nutrition level of experimental diets (as-fed basis)

| Items | Groups | | | | |
| --- | --- | --- | --- | --- | --- |
|  | Control group | Ⅰ | Ⅱ | Ⅲ | Ⅳ |
| Ingredients, % | | | | | |
| Corn | 68.61 | 62.00 | 55.00 | 48.00 | 41.00 |
| Wheat bran | 15.4 | 15.8 | 16.15 | 16.67 | 17.21 |
| Defatted rice bran (DFRB) | 0 | 7 | 14 | 21 | 28 |
| Soybean meal, 46% | 13.3 | 11.7 | 10.4 | 8.95 | 7.5 |
| Soybean oil | 0 | 0.84 | 1.83 | 2.78 | 3.74 |
| Lysine, 77.5% | 0.03 | 0.04 | 0.03 | 0.03 | 0.03 |
| Salt | 0.3 | 0.3 | 0.3 | 0.3 | 0.3 |
| Limestone | 0.82 | 0.85 | 0.85 | 0.85 | 0.85 |
| Calcium hydrogen phosphate | 0.75 | 0.68 | 0.65 | 0.63 | 0.58 |
| Choline, 50% | 0.04 | 0.04 | 0.04 | 0.04 | 0.04 |
| Premix^1^ | 0.4 | 0.4 | 0.4 | 0.4 | 0.4 |
| Nutrient level^2^ | | | | | |
| Dry matter, % | 88.56 | 88.68 | 88.93 | 89.16 | 88.46 |
| ME, MJ•kg^-1^ | 12.13 | 12.13 | 12.22 | 12.27 | 12.31 |
| Crude protien, % | 14.05 | 14.02 | 14.07 | 14.07 | 14.08 |
| Ether extract, % | 5.19 | 5.08 | 5.32 | 5.27 | 5.38 |
| Starch, % | 45.50 | 43.89 | 42.05 | 40.24 | 38.43 |
| Neutral detergent fiber, % | 8.89 | 11.8 | 12.93 | 14.35 | 17.94 |
| Acid detergent fiber, % | 5.53 | 6.25 | 6.53 | 7.08 | 8.13 |
| Insoluble dietary fiber, % | 16.14 | 17.19 | 18.42 | 19.32 | 23.37 |
| Soluble dietary fiber, % | 0.52 | 0.56 | 0.68 | 0.73 | 0.82 |
| Total dietary fiber, % | 16.7 | 17.75 | 19.1 | 20.05 | 24.11 |
| Cellulose, % | 4.06 | 4.43 | 4.71 | 5.09 | 5.79 |
| Hemicellulose, % | 3.37 | 5.55 | 6.4 | 7.28 | 9.81 |
| Lignin, % | 0.46 | 0.54 | 0.72 | 0.96 | 1.13 |
| Calcium, % | 0.55 | 0.55 | 0.55 | 0.55 | 0.55 |
| Available phosphorus, % | 0.27 | 0.27 | 0.27 | 0.27 | 0.27 |
| Standardized ileal digestible AA, %^3^ | | | | | |
| Lys | 0.59 | 0.57 | 0.55 | 0.53 | 0.52 |
| Met | 0.25 | 0.26 | 0.25 | 0.25 | 0.25 |
| Thr | 0.43 | 0.43 | 0.41 | 0.41 | 0.40 |
| Trp | 0.15 | 0.16 | 0.15 | 0.15 | 0.15 |
| Ile | 0.45 | 0.45 | 0.43 | 0.41 | 0.40 |
| Leu | 1.12 | 1.08 | 1.03 | 0.98 | 0.93 |
| Val | 0.58 | 0.58 | 0.57 | 0.56 | 0.55 |
| Arg | 0.78 | 0.78 | 0.77 | 0.77 | 0.76 |
| His | 0.37 | 0.37 | 0.35 | 0.34 | 0.33 |
| Phe | 0.53 | 0.52 | 0.50 | 0.48 | 0.46 |

^1^The premix was from Shanghai FuLangTe Animal Health Products Co., Ltd (Shanghai, China). The premix provided the following per kg of diets: vitamin D_3_ 1 500 IU, vitamin A 8 000 IU, vitamin K_3_ 4 mg, vitamin E 100 mg, vitaminB_2_ 8 mg, vitamin B_1_ 2 mg, vitamin B_12_ 0.04 mg, vitamin B_6_ 3 mg, pantothenic acid 35 mg, niacin 30 mg, biotin 0.13 mg, folic acid 0.6 mg, Choline 150 mg, Fe 60 mg, Zn 60 mg, Cu 5 mg, Se 0.15 mg, Mn 10 mg and I 0.1 mg.

^2^Dry matter, Ether extract, Neutral detergent fiber, Acid detergent fiber, Hemicellulose, cellulose, Lignin, Total dietary fiber, Insoluble dietary fiber and Soluble dietary fiber were measured values, while the other nutrients were calculated values.

^3^ Values for standardized ileal digestible (SID) concentrations of amino acids for the diets were estimated using standardized ileal digestible coefficients for the various ingredients provided by Nutrient Requirements of Swine (GB/T 39235-2020)

**Table S2** Analyzed chemical composition of the corn and defatted rice bran (DM basis)

| Items | Corn | Defatted rice bran | *P*-value |
| --- | --- | --- | --- |
| Crude protein, % | 10.62±0.12 | 18.54±0.18 | <0.000 |
| Ether extract, % | 5.49±0.15 | 3.13±0.21 | <0.000 |
| Crude fiber, % | 2.42±0.19 | 10.88±0.08 | <0.000 |
| Neutral detergent fiber, % | 11.12±0.25 | 30.09±0.47 | <0.000 |
| Acid detergent fiber, % | 3.24±0.10 | 11.39±0.08 | <0.000 |
| Cellulose, % | 2.93±0.11 | 6.84±0.45 | <0.000 |
| Hemicellulose, % | 7.82±0.21 | 18.44±0.46 | <0.000 |
| Lignin, % | 0.36±0.01 | 4.24±0.44 | <0.000 |
| Insoluble dietary fiber, % | 8.92±0.25 | 26.1±0.17 | <0.000 |
| Soluble dietary fiber, % | 0.79±0.11 | 1.36±0.20 | 0.027 |
| Total dietary fiber, % | 9.71±0.27 | 27.33±0.25 | <0.000 |

Note: All indicators are measured values.

**Table S3** PERMANOVA analysis of the factors affecting the fecal microbiota (multivariate models).

| Items | Sums of Sqs | Mean Sqs | *F* | *R2* | *P* |
| --- | --- | --- | --- | --- | --- |
| Average NDF intake | 0.49 | 0.49 | 5.13 | 0.18 | 0.002 |
| Average SDF intake | 0.38 | 0.38 | 3.77 | 0.14 | 0.004 |
| Average TDF intake | 0.37 | 0.37 | 3.64 | 0.13 | 0.004 |
| Average ADF intake | 0.38 | 0.38 | 3.81 | 0.14 | 0.004 |
| Average IDF intake | 0.37 | 0.37 | 3.68 | 0.13 | 0.005 |
| Average Cellulose intake | 0.36 | 0.36 | 3.57 | 0.13 | 0.006 |
| Average CP intake | 0.13 | 0.13 | 1.22 | 0.05 | 0.260 |
| Average EE intake | 0.13 | 0.13 | 1.14 | 0.05 | 0.268 |

**Table S4** Activity of fiber degrading enzyme analysis

| Items | Groups | | | | SEM | *P* value | |
| --- | --- | --- | --- | --- | --- | --- | --- |
|  | Control group | Ⅰ | Ⅱ | Ⅳ |  | Linear | Quadratic |
| Filter paper activity | 147.81 | 134.7 | 146.33 | 151.09 | 5.40 | 0.615 | 0.566 |
| Carboxymethyl cellulase | 90.83 | 89.55 | 96.84 | 101.36 | 1.95 | 0.023 | 0.833 |
| Salicinase | 78.61 | 79.62 | 73.85 | 77.30 | 1.25 | 0.513 | 0.382 |
| Microcrystalline cellulase | 92.74 | 90.24 | 107.51 | 104.65 | 2.30 | 0.009 | 0.316 |


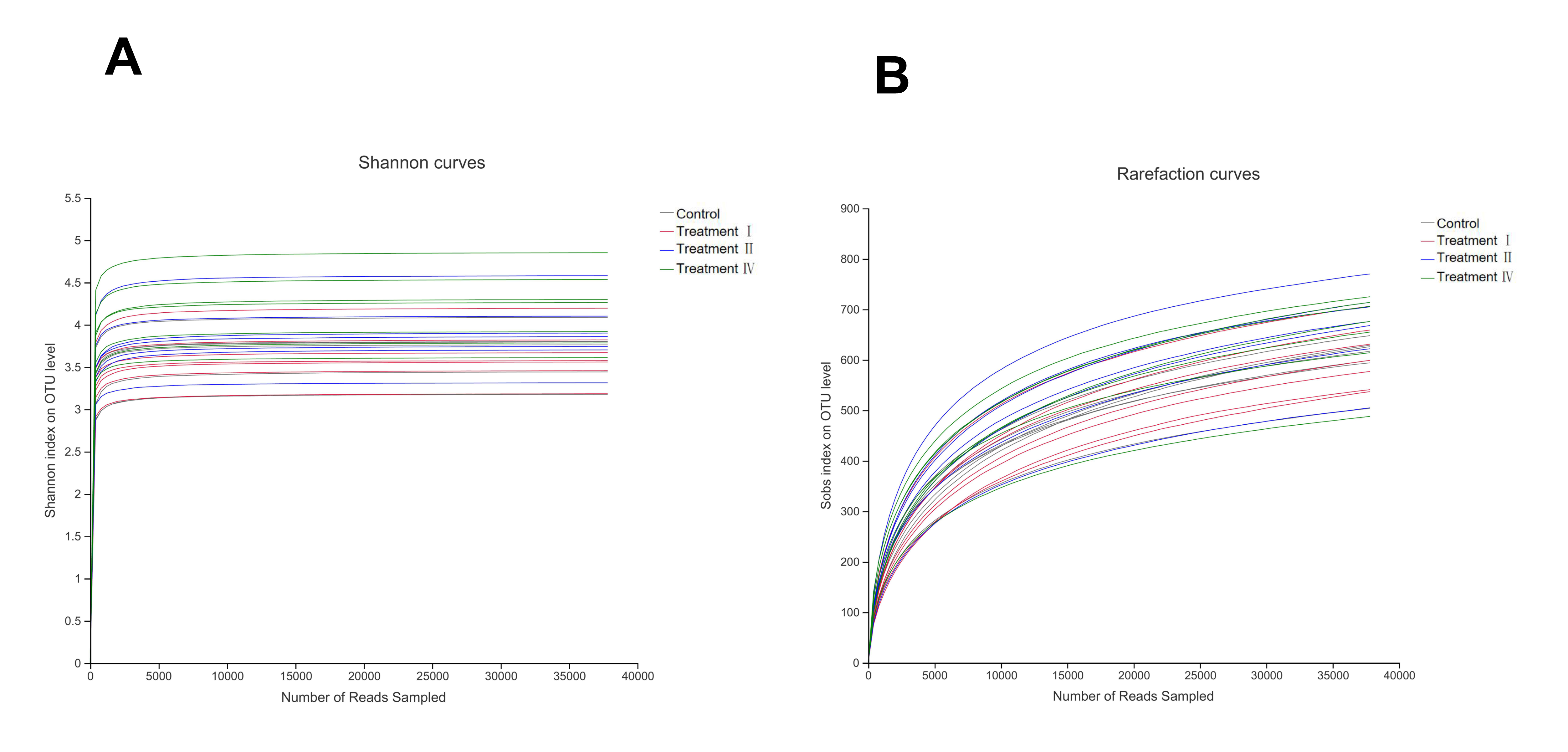


**Figure S1** The rarefaction curves in bacterial communities based on the Shannon index A) and the Sobs index B) in feces.
